# Investigation of Relationships between Urinary Biomarkers of Phytoestrogens, Phthalates, and Phenols and Pubertal Stages in Girls

**DOI:** 10.1289/ehp.0901690

**Published:** 2010-03-22

**Authors:** Mary S. Wolff, Susan L. Teitelbaum, Susan M. Pinney, Gayle Windham, Laura Liao, Frank Biro, Lawrence H. Kushi, Chris Erdmann, Robert A. Hiatt, Michael E. Rybak, Antonia M. Calafat, the Breast Cancer and Environment Research Centers

**Affiliations:** 1 Mount Sinai School of Medicine, New York, NY, USA; 2 University of Cincinnati College of Medicine, Cincinnati, Ohio, USA; 3 California Department of Public Health, Richmond, California, USA; 4 Cincinnati Children’s Hospital Medical Center, Cincinnati, Ohio; 5 Kaiser Permanente Division of Research, Oakland, California, USA; 6 Department of Epidemiology, University of Michigan, Ann Arbor, Michigan, USA; 7 Department of Epidemiology and Biostatistics, University of California San Francisco, San Francisco, California, USA; 8 National Center for Environmental Health, Centers for Disease Control and Prevention, Atlanta, Georgia, USA; 9 Breast Cancer and Environment Research Centers, http://www.bcerc.org/index.htm

**Keywords:** biomarkers, phenols, phthalates, phytoestrogens, puberty

## Abstract

**Background:**

Hormonally active environmental agents may alter the course of pubertal development in girls, which is controlled by steroids and gonadotropins.

**Objectives:**

We investigated associations of concurrent exposures from three chemical classes (phenols, phthalates, and phytoestrogens) with pubertal stages in a multiethnic longitudinal study of 1,151 girls from New York City, New York, greater Cincinnati, Ohio, and northern California who were 6–8 years of age at enrollment (2004–2007).

**Methods:**

We measured urinary exposure biomarkers at visit 1 and examined associations with breast and pubic hair development (present or absent, assessed 1 year later) using multivariate adjusted prevalence ratios (PR) and 95% confidence intervals (CIs). Modification of biomarker associations by age-specific body mass index percentile (BMI%) was investigated, because adipose tissue is a source of peripubertal hormones.

**Results:**

Breast development was present in 30% of girls, and 22% had pubic hair. High-molecular-weight phthalate (high MWP) metabolites were weakly associated with pubic hair development [adjusted PR, 0.94 (95% CI, 0.88–1.00), fifth vs. first quintile]. Small inverse associations were seen for daidzein with breast stage and for triclosan and high MWP with pubic hair stage; a positive trend was observed for low-molecular-weight phthalate biomarkers with breast and pubic hair development. Enterolactone attenuated BMI associations with breast development. In the first enterolactone quintile, for the association of high BMI with any development, the PR was 1.34 (95% CI, 1.23–1.45 vs. low BMI). There was no BMI association in the fifth, highest quintile of enterolactone.

**Conclusions:**

Weak hormonally active xenobiotic agents investigated in this study had small associations with pubertal development, mainly among those agents detected at highest concentrations.

Over the past 50 years, a trend has been reported toward earlier pubertal development in girls, with the implication that early maturation may lead to adverse social and medical conditions, including cancer and diabetes (Schoeters et al. 2008). Race, obesity, and genetics are likely determinants of pubertal timing, but hormonally active environmental exposures may also play a role (Jacobson-Dickman and Lee 2009). Widespread exposure exists to such environmental agents. Children and minorities often have higher exposures, as demonstrated by urinary concentrations of many environmental biomarkers, compared with adults and whites [Centers for Disease Control and Prevention (CDC) 2005]. Specific chemicals that behave like estradiol include a number of phenols, such as bisphenol A. They act as hormone agonists in animal models of reproductive development, accelerating pubertal development. However, phytoestrogens and phthalates have both agonist and antagonist effects in animals, likely related to alternative mechanisms, dose levels, and exposure timing (Rasier et al. 2006).

The Breast Cancer and Environment Research Centers (BCERC) are a consortium established by the National Institute of Environmental Health Sciences and The National Cancer Institute to elucidate influences of environmental factors on timing of pubertal development in girls. For this purpose, we evaluated exposure using concurrent urinary biomarkers representing three classes of environmental agents in relation to breast and pubic hair development among the girls in this cohort. Biomarkers were selected based on a pilot study that revealed a wide range of values and high detectability in our cohort (Wolff et al. 2007). We hypothesized that phenols would be associated with earlier puberty, that phthalate biomarkers would be related to later pubertal timing, that phytoestrogens would be associated with later breast development, and that associations could be modified by obesity.

## Materials and Methods

### Study population

The BCERC epidemiology project is a longitudinal study of girls enrolled at 6–8 years of age and followed through puberty. It is part of a consortium of four centers with transdisciplinary research collaborations integrated across biologic, epidemiologic, and community outreach projects. Enrollment of 1,239 girls during 2004–2007 occurred at three sites: Mount Sinai School of Medicine (MSSM), which recruited in East Harlem in New York City; Cincinnati Children’s Hospital (Cincinnati), which recruited in the greater Cincinnati, Ohio, metropolitan area, and through the Breast Cancer Registry of Greater Cincinnati; and Kaiser Permanente Northern California (KPNC), which recruited members of the KPNC Health Plan in the San Francisco Bay Area. All sites obtained informed consent from parent or guardian and independently verified child assent, approved by the institutional review boards at each institution and at the CDC. Eligibility included age, female sex, no underlying endocrine medical conditions, and at MSSM, black or Hispanic race/ethnicity.

### Data collection

A questionnaire was completed by the parent or guardian of the girl (usually the mother) that included medical history, product use and exposures, and demographic variables. Parents or guardians identified the girls as black, white, Asian, or other, and ethnicity as Hispanic or non-Hispanic. We assessed age, weight, height, breast and pubic hair stages at the visit when urine was collected (visit 1) and approximately 1 year later (visit 2). Visits 1 for MSSM and KPNC were at baseline. Visit 1 for Cincinnati is defined for this analysis as the visit when urine was collected, which was 6 months after the first baseline visit. Visit 2 was approximately 1 year later [details in Supplemental Material, Table 1 (doi:10.1289/ehp.0901690)]. At each visit, breast (B1–B5) and pubic hair (PH1–PH5) stages were assessed by inspection and palpation. Examiners were trained and tested by a master clinician, following a written protocol with photographs that demonstrated the maturation stages (Biro et al., in press; van Wieringen et al. 1985). Inter-rater evaluations were conducted by one pediatrician. The kappa statistic was 0.67, indicating substantial agreement; concordance was 87% (117/127 among 39 examiners) (Biro et al., in press). Height and weight were measured using calibrated scales and stadiometers at each visit. Age-specific (in months) and sex-specific body mass index percentiles (BMI%) were calculated based on CDC growth charts (CDC 2000). Pubertal stages and BMI% distribution at visit 1 were similar among girls with visit 2 breast stages (*n* = 985) and without (*n* = 166) visit 2 data; however, girls without visit 2 data were more likely to be black or Hispanic, of lower socioeconomic status, and from MSSM (Table 1).

### Urinary biomarker measurements

Samples collected at visit 1 were analyzed at the National Center for Environmental Health laboratories at CDC for nine phthalate metabolites [*n* = 1,149; monoethylphthalate (MEP), mono-butyl phthalate, mono-*iso*-butyl phthalate, mono-benzyl phthalate, mono-3-carboxypropyl phthalate, mono-2-ethyl-5-carboxypentyl phthalate, mono-(2-ethyl-5-hydroxylhexyl) phthalate, mono-(2-ethyl-5-oxohexyl) phthalate, and mono-(2-ethylhexyl) phthalate (MEHP)], seven phenols (benzophenone-3, bisphenol A, 2,5-dichlorophenol, triclosan; *n* = 1,149; methyl-, butyl-, and propyl- parabens, *n* = 1,059), and three phytoestrogens (daidzein, genistein, enterolactone; *n* = 1,150). Parabens were not measured early in the study. At least one urinary biomarker measurement was available among 1,151 girls, 985 with breast stages. We substituted limit of detection (
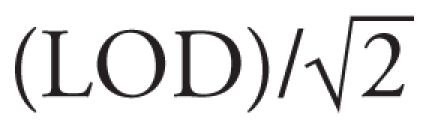
 for results below the LOD. Adjustment for urine dilution was accomplished using creatinine, to account for difference in sampling (spot specimens at MSSM and KPNC, early-morning samples at Cincinnati) and interindividual variation in urine dilution. We included log-creatinine in models using continuous log-biomarker variables, and we created quintile cut points from creatinine-corrected concentrations (micrograms per gram creatinine). As previously described, to reduce multiple comparisons we combined the phthalate metabolites into two groups that represent similar sources and similar biologic activity, low- (< 250 Da) and high-molecular-weight (> 250 Da) phthalate metabolites (low MWP and high MWP) [details in Supplemental Material, Table 2 (doi:10.1289/ehp.0901690)]. We expressed high MWP molar sum as MEHP (molecular weight 278) and the low MWP as MEP (molecular weight 194) so that units were the same as the other analytes (micrograms per liter). Similarly, a molar sum of the paraben metabolites was created (paraben sum) expressed as propyl paraben (molecular weight 180.2). Models with the individual phthalate and paraben metabolites were consistent with the molar sum variables. Results using di(2-ethylhexyl)phthalate (DEHP)-sum metabolites were almost identical to those for the high MWP, and they represented 75% ± 16% (mean ± SD) of the high MWP biomarkers. Therefore, only the latter models are presented.

Laboratory techniques and quality control protocols are identical to those reported previously in a pilot study (Wolff et al. 2007). Briefly, urine undergoes an automated cleanup with enzymatic deconjugation, followed by high-performance liquid chromatography-isotope dilution tandem mass spectrometry quantification (Kato et al. 2005; Rybak et al. 2008; Ye et al. 2005, 2006). In addition to the internal CDC quality control procedures, we incorporated approximately 10% masked quality control specimens (*n* = 101) from a single urine pool. The coefficients of variation (SD/mean concentration) were < 10% for 13 analytes and were between 10% and 21% for the remaining six biomarkers.

### Statistical analyses

We examined relationships among pubertal stages, biomarkers, and study characteristics using nonparametric statistics (Spearman or Kruskal–Wallis) and multivariate linear regression (version 9.1.3; SAS Institute Inc., Cary, NC). We conducted multivariate analyses using Proc Genmod (SAS) with modified Poisson regression, which provides robust error variance estimates and is appropriate for outcomes that are not rare (Zou 2004). We computed prevalence ratios (PRs) and 95% confidence intervals (CIs) for any development [breast stage 2 or higher (B2+); pubic hair stage 2 or higher (PH2+)] versus no development (B1 or PH1) in relation to biomarker exposures. We considered covariates related to pubertal development and urinary biomarker concentrations. Variables that did not alter any biomarker estimate by > 10% were backward-eliminated from the models. Excluding observations with very dilute biospecimens (*n* = 58 with creatinine < 0.2 g/L) did not materially change biomarker estimates; therefore, they were retained in the analyses. We assessed the trend of estimates for biomarker quintiles using their ordinal values with the contrast option in Proc Genmod. Models with continuous log-biomarker variable were consistent with results of models using quintiles. We report results for pubertal stages assessed at visit 2, one year after urine collection, when the proportions with B2+ and PH2+ were greater and site variations were less pronounced. There were 985 girls with biomarkers and visit 2 breast stage and 967 with visit 2 pubic hair stages.

We hypothesized that exogenous exposures were likely to operate jointly with BMI, a strong endogenous hormonal risk factor for pubertal development (Kaplowitz 2008). This hypothesis was examined by adding to models an interaction term for the product of the biomarker variable (log-continuous or ordinal quintile values) times BMI dichotomized at the median of the age-specific percentiles in our study population (low BMI/high BMI).

## Results

BCERC cohort girls were 6–8 years of age and mainly black, Hispanic, and white race/ethnicities (Table 1). Median ages differed by < 0.5 years across the sites, being youngest at MSSM and oldest at Cincinnati at both visits. Every site had > 20% blacks, but there were no whites from MSSM and very few Hispanics at Cincinnati. All characteristics including pubertal stages differed by site (Table 1; *p* < 0.01). Compared with normative national data for BMI, 65% of girls were above the 50th percentile at visit 1 (i.e., 15% more than expected), 32% were above the 85th percentile (overweight), and 17% were also above the 95th percentile (obese) (CDC 2000). BMI% was highest among MSSM (median 75th percentile of the norm) and lowest among Cincinnati girls (median 64th percentile).

As expected, the environmental biomarkers were detected in almost all of the urine samples [Supplemental Material, Table 2 (doi:10.1289/ehp.0901690)]. Overall, phenol median concentrations were < 100 μg/L, and urinary phytoestrogen and phthalate biomarker medians were > 400 μg/L. Geometric mean concentrations (micrograms per liter) of urinary exposure biomarkers are presented in Table 2 mutually adjusted for covariates. Benzophenone-3 (BP3), a sunscreen, was higher in samples collected in the summer and among white children, compared with blacks and Hispanics. 2,5-Dichlorophenol (2,5-DCP) was lower in whites and higher in MSSM participants; it is the metabolite of 1,4-dichlorobenzene used in mothballs and room deodorizers. Parabens are found as preservatives in many personal care products; their levels were higher among blacks and in samples collected in summer. Parabens often occur together with BP3 and low MWP in consumer products. Low-MWP biomarkers were higher in blacks and Hispanics than whites and Asians. The isoflavones daidzein and genistein, which are found in soy products, were somewhat higher among Asians. Enterolactone, a gut metabolite of lignans found in flax, beans, grains, and berries, tended to be higher among blacks and whites.

One year after urine samples were collected for biomarker measurements, breast development was present in 30% and pubic hair in 22% of girls (Table 1). As shown in Table 3, the frequency of any breast and pubic hair development increased or decreased across quintiles of most biomarkers, but the adjusted PRs were close to null. Low MWP had weak positive associations with both breast and pubic hair stages. For breast development, the adjusted PR was 1.06 (95% CI, 0.99–1.14; *p*-trend = 0.087) in the fifth versus first quintile. The isoflavones daidzein and genistein had weak inverse associations with breast stage; for daidzein, the adjusted PR was 0.96 for the fifth versus first quintile (95% CI, 0.90–1.02; *p*-trend = 0.061). Triclosan had a suggestive inverse association with pubic hair development, but the trend was not monotonic, and the CIs were similar for every quintile (Table 3). An inverse relationship with pubic hair development was seen for high MWP.

Enterolactone modified the association of BMI with breast development. The proportion of low-BMI girls at pubertal stages B2+ increased from 15 to 20% across enterolactone quintiles (see note to Table 4). Among girls with high BMI, the proportion in the first quintile (48% B2+) decreased to 25% B2+ in the fifth quintile. This inverse association among high-BMI girls supported our hypothesis, and it remained after further adjustment for covariates (Table 4). The difference in the enterolactone PRs between low- and high-BMI groups suggests that higher enterolactone exposures may attenuate the BMI association. Specifically, in the first quintile of enterolactone in Table 4, the PR for pubertal stage B2+ versus B1 was 1.34 (95% CI, 1.23–1.45) for high BMI compared with low-BMI girls, the referent. This difference, the “BMI association,” diminished steadily so that in the fifth quintile of enterolactone, the PRs for B2+ versus B1 were comparable for low BMI and high BMI (PR = 1.14 and 1.20, respectively, with similar CIs) (Table 4). Thus, breast development differed among low- and high-BMI girls with low enterolactone exposure, but development was similar at the highest exposure levels regardless of BMI. These enterolactone associations with breast stage were similar in models stratified by BMI and further adjusted for BMI%; results were also consistent by race/ethnicity and by site.

Apparent modification by BMI% of three other biomarkers in relation to pubertal stages could be explained by residual confounding of race or BMI% [*p*-interactions < 0.1; see Supplemental Material, Table 3 (doi:10.1289/ehp.0901690)]. In addition, genistein had a borderline interaction, but neither BMI stratum exhibited a trend of the adjusted PRs across biomarker quintiles. 2,5-DCP exhibited a positive association with breast development among high-BMI girls (*p*-interaction = 0.071; *n* = 948; *p*-trend 0.009), but in models stratified by BMI% further adjusted for BMI%, the association among high-BMI girls was null. The resulting attenuated estimate was similar to that of the main effect model for 2,5-DCP shown in Table 3. High MWP (*p*-interaction = 0.039) did not exhibit a dose–response relationship with breast development in low- or high-BMI groups (*p*-trends > 0.80). BP3 exhibited patterns similar to those of enterolactone. The BP3 association with breast stage had a positive direction in low-BMI girls and an inverse relationship in high-BMI girls (*p*-interaction 0.088). However, no dose–response relationship was evident. In BMI-stratified models, among girls with low-BMI the adjusted PR was 1.08 for the fifth versus first quintile of BP3 (95% CI, 0.97–1.20; *p*-trend 0.15; *n* =4 69); in high-BMI girls the *p*-trend was 0.38 (not shown).

## Discussion

In this group of 1,151 girls, we examined concurrent exposures from three chemical classes that possess known or likely hormonal activity in relation to pubertal development. Biologically relevant levels of the biomarkers existed among girls in the cohort. Most biomarkers were ubiquitously detected, and maximal concentrations were in the range known to elicit effects experimentally (e.g., > 10 μmol). Overall, biomarker concentrations were similar to those reported in the National Health and Nutrition Examination Survey (NHANES) for children 6–11 years of age (CDC 2005), as were those in the pilot study (Wolff et al. 2007). These urinary metabolites are derived from common personal and household products or dietary sources, and absorption may occur through ingestion, inhalation, or dermal routes (National Science Foundation 2008).

Associations of concurrent exposure biomarkers with breast and pubic hair development in these girls were not strong, but those observed were among the chemicals with greatest exposure levels. The strongest finding was attenuation by enterolactone exposure of the BMI association with breast development. Along with the inverse relationships of daidzein and genistein with breast development and high MWP with pubic hair stage, the results were consistent with our *a priori* hypotheses and the experimental literature. Comparable associations of phytoestrogens with breast stage were seen in an earlier small study (Wolff et al. 2008). Phytoestrogens including enterolactone are known to possess hormonal activity (Adlercreutz 2002). A protective effect for puberty might be consistent with counteracting the influence of obesity (Horn-Ross 1995) or by reducing adiposity (Cederroth et al. 2007). In contrast, associations were positive, albeit very weak, for low MWP with both breast and hair development. It is not clear why low- and high-MWP metabolites could have opposite associations with developmental stages, yet various reports of such exposures in humans and animals show divergent hormonal associations, depending on timing and intensity of exposure or treatment and on rodent strain (Adlercreutz 2002; Rasier et al. 2006; Schoeters et al. 2008; Shen et al. 2009). In addition, patterns and density of ambient exposures no doubt differ for the low MWPs and high MWPs (Adibi et al. 2008).

Residual confounding or misclassification of exposures and outcomes remain possible explanations for our results. Collinearity of covariates, such as that among BMI, race/ethnicity, urinary creatinine, and urinary biomarkers and their variation by study site, are potential difficulties. We used methods with robust variance handling in an effort to minimize such problems. A potential explanation for the lack of strong associations is overadjustment of the models due to the inclusion of certain covariates (Greenland et al. 1999; Weinberg 1993). For example, BMI may be both a confounder and on the pathway between exposure and pubertal development. Considering the interrelationships among our variables, the models presented are the most appropriate. For our main analyses, we used creatinine-corrected biomarker concentrations to create quintile cut points. Creatinine correction may be inadequate for some or all analytes. However, we did not measure specific gravity, an alternate measure of urine dilution (Hauser et al. 2004; Miller et al. 2004). Other methods, such as the regression normalization procedures (Heavner et al. 2006), may not be appropriate for all urinary metabolites. Besides exposure misclassification, there is potential error in the outcome measurement of pubertal stage because of inter-rater variability in pubertal stage assessment. Therefore, both exposure and outcome measurements may be subject to nondifferential misclassification bias, which would likely shift the estimates toward the null. Additional considerations, including genetic and racial differences in exposure and development, would require considerably larger sample size. We estimated that for the main effects we had adequate power (80%) to detect PRs of 1.1 in 479 girls, if B2+ or PH2+ were > 20% in the fifth quintile (alpha 0.05), and a PR of 0.94 with 948 girls; these values are similar to the strongest associations we observed. Our effect estimates also may be conservative, because we used Poisson models instead of logistic regression models. For example, for the PR of 0.94 (95% CI, 0.88–1.00, high MWP and PH2+) in Table 3, we computed an odds ratio (OR) of 0.60 (95% CI, 0.34–1.06). However, measures of association using ORs may be over- or underestimated (Zou 2004). Finally, some or all of our findings may be due to chance; > 100 comparisons were made for the models presented in Tables 3 and 4. Associations of hormonal exposures in this study were small, which may be consistent with their relatively weak biological activity compared with endogenous hormones (Fang et al. 2000; Shen et al. 2009). Small effect estimates may be more realistic than those in previous studies that had small sample sizes (Wolff et al. 2008). There will be greater power to detect associations in longitudinal analyses that can also better reflect causal relationships than cross-sectional analyses; we plan to undertake such analyses as the cohort matures. The reports of delayed pubertal development in relation to blood lead concentrations in the NHANES population are informative for our findings, as the lead effects appear stronger than those we observed. Selevan et al. (2003) observed among black girls an OR of 0.62 (95% CI, 0.41–0.96, multivariate adjusted) for PH2+ versus PH1 among girls with blood lead > 3 μg/dL, quartile of exposure, compared with those having blood lead < 1 μg/dL, approximately the upper versus first quartile of their exposures. For the same NHANES population, Wu et al. (2003) found ORs for PH2+ of 0.27 (95% CI, 0.08–0.93) in the top exposure group (≥ 5 μg/dL) compared with ≤ 2 μg/dL blood lead. The proportion of PH2+ in the low exposure stratum was 81% versus 44% at high exposure. By comparison, the prevalence of PH2+ was 28% in the first compared with 20% in the fifth quintile of high MWP in Table 3, and the adjusted PR was 0.94 (95% CI, 0.88–1.00). High-MWP medians were 7-fold different between these quintiles compared with 3-fold differences in the lead exposure categories.

An additional consideration is that the peripubertal period is likely not the only critical window of exposure for pubertal development. Both animal and epidemiology studies suggest that prenatal and perinatal exposures also exert effects on later development (Rasier et al. 2006; Schoeters et al. 2008). Exposures during different windows may affect different molecular targets, including prenatal imprinting, the hypothalamic-pituitary axis, gonadotropin-releasing hormone neurons, hormone receptors, and aromatase action (Schoeters et al. 2008). Effective exposure ranges for these mechanisms may also differ widely, that is, toxic equivalents. Environmental agents in our study are cleared rapidly; it is possible that a single biomarker measurement is inadequate to quantify exposure relevant to pubertal development. However, single measurements of urinary biomarkers of phenols and phthalates were fairly representative of 6–12 months of exposure in children this age (Teitelbaum et al. 2008), likely because of common use and continuous exposure to many chemicals. Time-integrated multiple childhood exposure measures prenatally and prepubertally may be possible in alternate study designs. An important additional direction is to evaluate multiple exposures, including the extremes of exposure, multiple high exposures, early life exposures, and/or extremes of development (very late vs. very early changes) (Chou et al. 2009).

As we have mentioned, these environmental biomarkers were considered important for pubertal development because their concentrations are higher and in some cases their bioassay potency is greater than commonly studied environmental agents such as lead and 1,1′-dichloro-2,2′-bis(4-chlorophenyl)ethylene (DDE). Although the suggestive associations we observed are small, within 10% of null, a small effect size could affect a significant proportion of the population because of the ubiquity of these exposures and by their high levels (micromolar) observed in urine of the BCERC cohort.

## Figures and Tables

**Table 1:** Characteristics at visit 1 and by site for 1,151 girls with at least one biomarker value: BCERC cohort, 2004–2007 [*n* (%)].

Variable	All sites combined	MSSM	Cincinnati	KPNC
Age at baseline (visit 1) (years)				
6.0–6.9	293 (25.5)	153 (37.7)	71 (22.0)	69 (16.4)
7.0–7.9	571 (49.6)	131 (32.3)	148 (45.8)	292 (69.2)
≥ 8.0–9.4	287 (24.9)	122 (30.0)	104 (32.2)	61 (14.5)
Median age	7.50	7.25	7.64	7.41

Age at visit 2 (years)				
6.9–7.9	245 (21.3)	105 (25.9)	65 (20.2)	75 (17.8)
8.0–8.9	478 (41.5)	97 (23.9)	133 (14.3)	247 (58.7)
≥ 9.0–10.6	266 (23.1)	103 (25.4)	92 (28.6)	70 (16.6)
Not seen including missing (1)	162	101	32	29
Median age	8.50	8.41	8.66	8.50

Child race/ethnicity				
White	391 (34.0)	0 (0.0)	220 (68.1)	171 (40.5)
Black	353 (30.7)	164 (40.4)	97 (30.0)	92 (21.8)
Hispanic, non-black	344 (29.9)	242 (59.6)	1 (0.3)	101 (23.9)
Asian	53 (4.6)	0 (0.0)	4 (1.2)	49 (11.6)
Other	10 (0.9)	0 (0.0)	1 (0.3)	9 (2.1)

BMI age- and sex-specific percentile				
< 50th	394 (34.2)	133 (32.8)	101 (31.3)	160 (37.9)
50–85th	388 (33.7)	114 (28.1)	132 (40.9)	142 (33.6)
≥ 85th	369 (32.1)	159 (39.2)	90 (27.9)	120 (28.4)

Parent or guardian education				
≤ High school	342 (29.7)	236 (58.1)	29 (9.0)	77 (18.2)
> High school	765 (66.5)	162 (39.9)	261 (80.8)	342 (81.0)
Missing	44	8	33	3

Season of urine collection				
Spring (March–May)	393 (34.1)	112 (27.6)	163 (50.5)	118 (28.0)
Summer (June–August)	322 (28.0)	94 (23.2)	48 (14.9)	71 (16.8)
Fall (September–November)	213 (18.5)	90 (22.2)	64 (19.8)	168 (39.8)
Winter (December–February)	223 (19.4)	110 (27.1)	48 (14.9)	65 (15.4)

Interval between visits 1 and 2				
< 14 months	893 (90.5)	239 (78.4)	288 (99.0)	367 (93.6)
≥ 14 months	94 (9.5)	66 (21.6)	3 (1.0)	25 (6.4)

Breast stage at visit 1				
B1	943 (81.9)	317 (78.1)	237 (73.4)	389 (92.2)
B2+	206 (17.9)	89 (21.9)	86 (26.6)	31 (7.4)
Missing	2	0	0	2

Breast stage at visit 2				
B1	688 (59.8)	194 (47.8)	167 (51.7)	327 (77.5)
B2+	297 (25.8)	111 (27.3)	124 (38.4)	62 (14.7)
Not seen including missing (5)	166	101	32	33

Pubic hair stage at visit 1				
PH1	979 (85.1)	334 (82.3)	275 (85.1)	370 (87.7)
PH2+	151 (13.1)	71 (17.5)	43 (13.3)	37 (8.8)
Missing	21	1	5	15

Pubic hair stage at visit 2				
PH1	756 (65.7)	216 (53.2)	230 (71.2)	310 (73.5)
PH2+	211 (18.3)	88 (21.7)	61 (18.9)	62 (14.7)
Not seen including missing (23)	184	102	32	50

Total *n*	1,151	406	323	422

**Table 2:** Geometric means (95% CIs) of environmental urinary biomarkers at baseline examination in relation to covariates, mutually adjusted and for ln-urinary creatinine (1,102 girls with all characteristics, 2004–2007).

	Phenols (μg/L)					Phthalate monoesters, μg/L		Phytoestrogens (μg/L)		
	BP3	BPA	2,5-DCP	Triclosan	Paraben sum	Low MWP	High MWP	Daidzein	Genistein	Enterolactone
Age at baseline exam (years)										
6.0–6.9	33 (24–45)	2.1 (1.8–2.4)	13 (10–17)	17 (13–23)	78 (61–101)	169 (145–199)	183 (158–212)	143 (106–194)	66 (49–90)	307 (248–381)
7.0–7.9	28 (21–37)	1.9 (1.7–2.2)	14 (11–18)	17 (13–23)	63 (50–79)	161 (140–186)	178 (156–202)	106 (81–139)	49 (37–64)	323 (267–390)
≥ 8.0	28 (20–38)	1.8 (1.5–2.1)	14 (11–19)	17 (13–23)	63 (48–81)	156 (133–182)	157 (136–182)	112 (83–151)*	50 (37–67)*	320 (259–396)

Race/ethnicity										
White	51 (41–63)	2.0 (1.8–2.3)	6.0 (5.1–7.5)	14 (11–17)	39 (33–47)	114 (102–127)	179 (162–199)	118 (96–146)	56 (45–69)	396 (341–461)
Black	12 (10–15)	2.2 (2.0–2.4)	22 (18–26)	16 (13–19)	170 (143–201)	211 (190–233)	150 (137–165)	96 (79–116)	40 (33–49)	351 (306–403)
Hispanic, non-black	22 (17–27)	2.1 (1.9–2.3)	20 (17–24)	15 (12–19)	83 (69–99)	190 (170–212)	178 (160–197)	80 (65–98)	38 (31–47)	273 (235–318)
Asian	32 (20–52)	1.5 (1.2–2.0)	14 ( 9.0–21)	14 (9.1–23)	55 (37–82)	117 (91–149)	174 (139–219)	203 (127–324)	118 (74–188)	269 (193–375)
Other	52 (17–160)**	1.9 (1.1–3.3)	15 (5.5–38)**	32 (11–90)	46 (19–113)**	211 (120–369)**	182 (109–305)*	133 (46–384)**	47 (16–137)**	311 (146–663)**

BMI age-specific percentile at baseline										
< 50th	31 (23–42)	2.0 (1.7–2.3)	13 (9.8–16)	19 (15–26)	70 (55–89)	153 (132–178)	180 (157–206)	116 (87–153)	53 (40–71)	376 (308–460)
50–85th	27 (20–36)	2.0 (1.7–2.3)	14 (11–18)	15 (12–20)	61 (48–78)	157 (136–182)	167 (146–192)	121 (91–161)	52 (39–69)	345 (282–421)
≥ 85th	31 (23–42)	1.9 (1.6–2.2)	15 (12–20)	17 (13–22)	72 (57–93)	177 (152–206)	170 (148–196)	121 (91–162)	58 (43–77)	245 (199–301)**

Caregiver education										
12 years	24 (18–33)	1.9 (1.6–2.2)	15 (11–20)	18 (14–24)	63 (49–81)	174 (149–203)	178 (154–205)	117 (87–157)	55 (41–74)	288 (233–356)
≥ 13 years	36 (28–47)**	2 (1.7–2.3)	13 (10–17)	16 (13–21)	72 (58–90)	151 (132–173)*	167 (147–189)	122 (95–158)	54 (42–70)	347 (289–417)*

Study site										
MSSM	22 (16–30)	2.3 (1.9–2.7)	46 (34–60)	15 (11–20)	70 (54–92)	201 (171–237)	242 (209–281)	89 (65–120)	42 (31–57)	287 (230–357)
Cincinnati	20 (14–28)	1.9 (1.6–2.2)	13 (9.6–17)	28 (21–39)	66 (50–88)	163 (138–194)	154 (131–180)	135 (97–186)	60 (43–82)	252 (200–318)
KPNC	58 (44–78)**	1.7 (1.5–2.0)**	4.7 (3.7–6.1)**	12 (9.0–16)**	66 (52–83)	129 (112–149)**	137 (120–157)**	143 (109–188)**	64 (49–84)*	439 (361–534)**

Season of urine collection										
Spring	20 (15–27)	1.8 (1.5–2.0)	13 (10–17)	17 (13–22)	66 (51–84)	154 (132–179)	169 (147–194)	134 (101–178)	64 (48–85)	310 (253–380)
Summer	125 (92–172)	2.4 (2.1–2.8)	13 (10–17)	18 (14–24)	93 (72–121)	208 (177–243)	185 (160–214)	98 (73–132)	45 (33–60)	271 (219–335)
Fall	22 (16–30)	2.0 (1.7–2.4)	18 (14–24)	15 (11–21)	54 (41–70)	145 (123–171)	175 (151–204)	124 (91–170)	57 (42–77)	349 (280–436)
Winter	14 (10–19)**	1.6 (1.4–2.0)**	13 (9.9–18)*	18 (13–25)	63 (48–83)**	149 (126–176)**	161 (138–188)	125 (91–171)	55 (40–76)*	343 (273–430)

When any adjusted geometric mean differed from one or more of the others within a characteristic, the bottom value is marked with an asterisk. BMI% is sex- and age-specific (CDC 2000). Spring is March, April, May; other seasons follow sequentially. Paraben sum is the molar sum of methyl-, butyl-, and propyl-parabens, expressed as propylparaben (MW 180.2). Low MWP is the molar sum of MEP, MBP (monobutyl phthalate), MIBP expressed as MEP (MW = 194). High MWP is the molar sum of MBZP (mono-benzyl phthalate), MCPP [mono(3-carboxypropyl) phthalate], MEHP, MEOHP [mono-(2-ethyl-5-oxohexyl) phthalate], MEHHP [mono-(2-ethyl-5-hydroxyhexyl) phthalate], and MECPP [mono(2-ethyl-5-carboxypentyl) phthalate] expressed as MEHP (MW = 278). DEHP metabolite sum, which was 75 ± 16% of high MWP and had almost identical means, is omitted from the table. Details are in Supplemental Material, Table 2 (doi:10.1289/ehp.0901690).

* *p* < 0.05,

** *p* < 0.01.

**Table 3:** PRs and 95% CIs for breast development stage (B2+ vs. B1) and pubic hair stage (PH2+ vs. PH1) in relation to urinary environmental biomarkers measured 1 year earlier. BCERC Cohort, 2004–2008.

	Quintiles of creatinine-corrected biomarker concentrations (C)					
	1st	2nd	3rd	4th	5th	*p*-Trend
Breast development						
Phenols						
BP3						
Quintile median μg/gC	3.6	11.1	30.4	93.6	867	
*n* B2+/*n* total	76/196	65/197	64/196	52/197	40/196	
PR (95% CI)	1 (Ref)	0.96 (0.89–1.03)	0.96 (0.89–1.02)	0.91 (0.85–0.98)	0.87 (0.81–0.93)	< 0.0001
Adj PR (95% CI)	1 (Ref)	1.00 (0.94–1.06)	1.01 (0.95–1.07)	0.99 (0.93–1.06)	1.01 (0.94–1.09)	0.847
BPA						
Quintile median μg/gC	1.0	1.6	2.4	3.7	8.7	
*n* B2+/total	69/196	59/197	54/196	61/197	54/196	
PR (95% CI)	1 (Ref)	0.96 (0.90–1.03)	0.94 (0.88–1.01)	0.97 (0.90–1.04)	0.94 (0.88–1.01)	0.171
Adj PR (95% CI)	1 (Ref)	0.96 (0.90–1.02)	0.95 (0.89–1.01)	0.97 (0.91–1.03)	0.97 (0.91–1.03)	0.533
2,5-DCP						
Quintile median μg/gC	1.6	4.0	10.7	37.2	179	
*n* B2+/total	34/196	51/197	62/196	74/197	76/196	
PR (95% CI)	1 (Ref)	1.07 (1.00–1.15)	1.12 (1.05–1.20)	1.17 (1.10–1.25)	1.18 (1.11–1.26)	< .0001
Adj PR (95% CI)	1 (Ref)	0.99 (0.93–1.06)	0.97 (0.91–1.04)	1.04 (0.96–1.12)	1.02 (0.94–1.10)	0.416
Triclosan						
Quintile median μg/gC	2.6	6.9	15	38	170	
*n* B2+/total	57/196	49/197	59/196	65/197	67/196	
PR (95% CI)	1 (Ref)	0.97 (0.90–1.04)	1.01 (0.94–1.08)	1.03 (0.96–1.10)	1.04 (0.97–1.11)	0.077
Adj PR (95% CI)	1 (Ref)	0.99 (0.93–1.05)	0.97 (0.92–1.03)	1.01 (0.95–1.07)	1.03 (0.97–1.10)	0.242
Paraben sum						
Quintile median μg/gC	15	36	84	198	839	
*n* B2+/total	45/180	50/181	58/181	45/181	68/180	
PR (95% CI)	1 (Ref)	1.02 (0.95–1.10)	1.06 (0.98–1.14)	1.00 (0.93–1.07)	1.10 (1.03–1.18)	0.036
Adj PR (95% CI)	1 (Ref)	1.01 (0.95–1.07)	1.02 (0.96–1.09)	0.98 (0.92–1.04)	1.03 (0.96–1.10)	0.653

Phthalates						
High MWP						
Quintile median μg/gC	84	138	204	326	616	
*n* B2+/total	67/196	62/197	53/196	40/197	75/196	
PR (95% CI)	1 (Ref)	0.98 (0.91–1.05)	0.95 (0.88–1.02)	0.90 (0.84–0.96)	1.03 (0.96–1.10)	0.716
Adj PR (95% CI)	1 (Ref)	0.98 (0.92–1.04)	0.98 (0.92–1.04)	0.94 (0.88–1.00)	1.03 (0.97–1.10)	0.781
Low MWP						
Quintile median μg/gC	66	112	173	272	721	
*n* B2+/total	46/196	54/197	55/196	64/197	78/196	
PR (95% CI)	1 (Ref)	1.03 (0.96–1.11)	1.04 (0.97–1.11)	1.07 (1.00–1.15)	1.13 (1.06–1.21)	0.000
Adj PR (95% CI)	1 (Ref)	1.03 (0.97–1.09)	1.01 (0.95–1.07)	1.04 (0.98–1.11)	1.06 (0.99–1.14)	0.087

Phytoestrogens						
Daidzein						
Quintile median μg/gC	20	48	104	281	1,359	
*n* B2+/total	59/196	65/197	67/197	58/196	47/196	
PR (95% CI)	1 (Ref)	1.02 (0.95–1.10)	1.03 (0.96–1.10)	1.00 (0.93–1.07)	0.95 (0.89–1.02)	0.121
Adj PR (95% CI)	1 (Ref)	1.05 (0.99–1.11)	1.02 (0.96–1.08)	1.00 (0.94–1.06)	0.96 (0.90–1.02)	0.061
Genistein						
Quintile median μg/gC	9.4	20	45	127	610	
*n* B2+/total	62/196	65/197	65/196	54/197	50/196	
PR (95% CI)	1 (Ref)	1.01 (0.94–1.08)	1.01 (0.94–1.08)	0.97 (0.90–1.04)	0.95 (0.89–1.02)	0.081
Adj PR (95% CI)	1 (Ref)	1.02 (0.96–1.08)	1.00 (0.94–1.06)	0.97 (0.91–1.03)	0.97 (0.92–1.03)	0.103
Enterolactone						
Quintile median μg/gC	97	283	522	899	1,922	
*n* B2+/total	66/196	62/197	66/196	57/197	45/196	
PR (95% CI)	1 (Ref)	0.98 (0.92–1.05)	1.00 (0.93–1.07)	0.96 (0.90–1.03)	0.92 (0.86–0.99)	0.018
Adj PR (95% CI)	1 (Ref)	1.02 (0.96–1.08)	1.03 (0.97–1.09)	1.00 (0.94–1.07)	1.03 (0.97–1.10)	0.462

Pubic hair development						
Phenols						
BP3						
Quintile median μg/gC	3.7	11	30	94	853	
*n* PH2+/*n* total	48/195	54/195	40/191	35/194	33/189	
PR (95% CI)	1 (Ref)	1.02 (0.96–1.10)	0.97 (0.91–1.04)	0.95 (0.89–1.01)	0.94 (0.88–1.01)	0.01
Adj PR (95% CI)	1 (Ref)	1.06 (1.00–1.14)	1.02 (0.96–1.09)	1.02 (0.95–1.09)	1.06 (0.98–1.15)	0.40
BPA						
Quintile median μg/gC	1.0	1.6	2.4	3.8	8.8	
*n* PH2+/total	39/194	44/196	52/190	38/191	37/193	
PR (95% CI)	1 (Ref)	1.02 (0.95–1.09)	1.06 (0.99–1.14)	1.00 (0.93–1.07)	0.99 (0.93–1.06)	0.67
Adj PR (95% CI)	1 (Ref)	1.03 (0.96–1.09)	1.06 (0.99–1.13)	0.99 (0.92–1.05)	1.00 (0.94–1.07)	0.57
2,5-DCP						
Quintile median μg/gC	1.6	4.0	11	37	179	
*n* PH2+/total	32/189	32/192	40/195	57/194	49/194	
PR (95% CI)	1 (Ref)	1.00 (0.94–1.06)	1.03 (0.97–1.10)	1.11 (1.03–1.18)	1.07 (1.00–1.15)	0.00
Adj PR (95% CI)	1 (Ref)	0.95 (0.89–1.02)	0.95 (0.89–1.02)	1.00 (0.92–1.08)	0.93 (0.86–1.01)	0.40
Triclosan						
Quintile median μg/gC	2.6	6.9	15	38	170	
*n* PH2+/total	56/191	37/192	44/192	32/195	41/194	
PR (95% CI)	1 (Ref)	0.92 (0.86–0.99)	0.95 (0.89–1.02)	0.90 (0.84–0.96)	0.94 (0.87–1.00)	0.06
Adj PR (95% CI)	1 (Ref)	0.93 (0.87–0.99)	0.93 (0.87–0.99)	0.89 (0.83–0.94)	0.94 (0.88–1.00)	0.02
Paraben sum						
Quintile median μg/gC	15	436	84	197	837	
*n* PH2+/total	33/178	25/177	37/178	44/174	50/179	
PR (95% CI)	1 (Ref)	0.96 (0.90–1.03)	1.02 (0.95–1.09)	1.06 (0.98–1.13)	1.08 (1.01–1.16)	0.00
Adj PR (95% CI)	1 (Ref)	0.94 (0.89–1.01)	0.95 (0.89–1.02)	1.02 (0.96–1.10)	0.97 (0.90–1.04)	0.80

Phthalates						
High MWP						
Quintile median μg/gC	84	138	203	327	613	
*n* PH2+/total	55/193	44/195	37/191	35/192	39/193	
PR (95% CI)	1 (Ref)	0.95 (0.89–1.02)	0.93 (0.87–0.99)	0.92 (0.86–0.98)	0.94 (0.87–1.00)	0.04
Adj PR (95% CI)	1 (Ref)	0.98 (0.92–1.04)	0.95 (0.90–1.01)	0.95 (0.89–1.01)	0.94 (0.88–1.00)	0.04
Low MWP						
Quintile median μg/gC	66	112	173	272	718	
*n* PH2+/total	30/190	30/192	50/194	44/195	56/193	
PR (95% CI)	1 (Ref)	1.00 (0.94–1.06)	1.09 (1.02–1.16)	1.06 (0.99–1.13)	1.11 (1.04–1.19)	0.00
Adj PR (95% CI)	1 (Ref)	1.00 (0.94–1.06)	1.05 (0.98–1.12)	1.03 (0.97–1.10)	1.06 (0.98–1.13)	0.08

Phytoestrogens						
Daidzein						
Quintile median μg/gC	20	48	104	281	1,347	
*n* PH2+/total	49/195	46/192	39/196	31/190	44/191	
PR (95% CI)	1 (Ref)	0.99 (0.92–1.06)	0.96 (0.90–1.02)	0.93 (0.87–0.99)	0.98 (0.92–1.05)	0.18
Adj PR (95% CI)	1 (Ref)	1.01 (0.95–1.08)	0.97 (0.91–1.03)	0.95 (0.89–1.01)	1.00 (0.94–1.07)	0.39
Genistein						
Quintile median μg/gC	9.5	20	46	129	607	
*n* PH2+/total	48/193	45/194	45/193	26/194	45/190	
PR (95% CI)	1 (Ref)	0.99 (0.92–1.06)	0.99 (0.92–1.06)	0.91 (0.85–0.97)	0.99 (0.92–1.06)	0.19
Adj PR (95% CI)	1 (Ref)	1.00 (0.94–1.06)	1.01 (0.95–1.07)	0.93 (0.88–0.99)	1.03 (0.96–1.09)	0.76
Enterolactone						
Quintile median μg/gC	97	282	522	899	1,832	
*n* PH2+/total	45/195	37/193	48/194	40/195	39/187	
PR (95% CI)	1 (Ref)	0.97 (0.91–1.04)	1.01 (0.95–1.09)	0.98 (0.92–1.05)	0.98 (0.92–1.05)	0.77
Adj PR (95% CI)	1 (Ref)	0.99 (0.93–1.05)	1.02 (0.96–1.08)	0.98 (0.92–1.05)	1.03 (0.96–1.10)	0.47

Abbreviations: Adj, adjusted; C, creatinine; Ref, referent. Adjusted models included age, race/ethnicity, sex- and age-specific BMI% (CDC 2000), guardian education, season of urine collection, and site. There were fewer paraben measurements than other biomarkers because they were not measured early in the study. Quintile cut points were based on 982 girls with breast stage data, biomarkers, and creatinine. Quintile medians are among girls with breast or pubic stages. Breast stages were available for 981 girls with all biomarkers (963 for pubic hair); one additional girl with phthalate/phenol biomarkers was B1, PH2+ (982 total breast, 964 PH); one additional girl with phytoestrogens was B2+, PH1 (982 total breast, 964 PH stages).

**Table 4:** Adjusted PRs (95% CIs) for any breast development stage (B2+ vs. B1) at visit 2 in relation to urinary environmental biomarkers and age-specific BMI measured at visit 1.

	Urinary enterolactone quintile [median creatinine (C) corrected biomarker concentration (μg/gC) for quintile]					
	1st (97 μg/gC)	2nd (283 μg/gC)	3rd (522 μg/gC)	4th (899 μg/gC)	5th (1,922 μg/gC)	*p*-Trend
*n* B2+ / *n* total	12/84	14/82	17/99	20/102	27/124	
Adjusted PR (95% CI) among low-BMI girls (BMI% range, < 1 to 68%; median = 36.0%)	1.0 (ref)	1.06 (0.96–1.15)	1.05 (0.96–1.15)	1.11 (1.01–1.22)	1.15 (1.05–1.26)	0.0016
*n* B2+ / *n* total	54/112	48/115	49/97	37/95	18/72	
Adjusted PR (95% CI) among high-BMI girls (BMI% range, 68.5 to > 99%; median = 90.1%)	1.34 (1.23–1.45)	1.30 (1.20–1.42)	1.35 (1.24–1.48)	1.24 (1.13–1.36)	1.21 (1.10–1.33)	0.0105

PRs were computed from model with interaction term (ordinal values of enterolactone biomarker quintiles times 2-level BMI), adjusted for age at visit 2, race/ethnicity, site, caregiver education, season of urine collection. *p*-Interaction = 0.0006.

## Appendix 1

We greatly appreciate the advice, support and collaboration of colleagues at NIH, including G. Collman, D. Winn, E. Maull, L. Reinlib, S. Lynch, G. Ellison, and C. Dilworth. We acknowledge the Avon Foundation for support and donations. We thank the study investigators and staff at the three medical centers involved in this research including M. Galvez, B. Brenner, J. Wetmur, J. Chen, P. Sheffield, N. Vangeepurum, J. Forman, L. Boguski, J. Britton, S. Peter, A. Mejia, A. Richiez, J. Montana, E. Chae, R. Osborne, E. Moshier, C. Zhu, and K. McGovern (MSSM); C. Dahl, C. Baker, S. Myatt, K. Ford, B. Bornschein, L. Yaghjyan, S. Roda (Cincinnati); L. Greenspan, B. Sternfeld, C. Ambrosone, J. Deardorff, S. Stewart, K. Balke, C.Ashley, C.Bonnell, A. Beeck, C. Chan, D. Davis, E. Landaverde, S. Burleson, R. Lum, A. Mirabedi, and M. Trotter (Kaiser Permanente). We are grateful to CDC colleagues M. Silva, E. Samandar, J. Preau, X. Ye, A. Bishop, J. Reidy, C. Pfeiffer, D. Parker, P. Olive, M. Kimberly, and C. Dodson.

Fox Chase Cancer Center and Mount Sinai acknowledge their community clinical collaborators, including North General Pediatric Clinic, Settlement Health Center, Children’s Aid Society, Little Sisters of the Assumption, Mount Sinai Pediatric Clinic, and our Community Outreach and Translation Core (COTC) partners (L. Claudio, S. Williams, D. Duncan, A. Fonfa). UCSF/Kaiser Permanente acknowledges their institutional and community collaborators, including Marin Breast Cancer Watch, California Department of Public Health, Marin County Department of Health and Human Services, San Francisco Department of Public Health, University of California Davis, University of Michigan, Roswell Park Cancer Institute, and our COTC partners (J. Barlow, K. Koblick). University of Cincinnati/Cincinnati Children’s Hospital acknowledges institutional and community collaborators, including the Breast Cancer Alliance of Greater Cincinnati, the Children’s Hospital Medical Center Clinical Research Center, the Breast Cancer Registry of Greater Cincinnati, Winton Woods School District, Fort Thomas School District, and our COTC partners (K. Brown, K. Ball).

## References

[b1-ehp-118-1039] Adibi JJ, Whyatt RM, Williams PL, Calafat AM, Camann D, Herrick R (2008). Characterization of phthalate exposure among pregnant women assessed by repeat air and urine samples. Environ Health Perspect.

[b2-ehp-118-1039] Adlercreutz H (2002). Phytoestrogens and breast cancer. J Steroid Biochem Mol Biol.

[b3-ehp-118-1039] Biro FM, Galvez M, Greenspan L, Succop P, Vangeepuram N, Pinney S Pubertal assessment methodology and baseline characteristics in a mixed longitudinal study of girls. Pediatrics.

[b4-ehp-118-1039] CDC (Centers for Disease Control and Prevention) (2000). CDC Growth Charts: United States.

[b5-ehp-118-1039] CDC (Centers for Disease Control and Prevention) (2005). National Report on Human Exposure to Environmental Chemicals.

[b6-ehp-118-1039] Cederroth CR, Vinciguerra M, Gjinovci A, Kühne F, Klein M, Cederroth M (2007). A phytoestrogen-rich diet increases energy expenditure and decreases adiposity in mice. Environ Health Perspect.

[b7-ehp-118-1039] Chou YY, Huang PC, Lee CC, Wu MH, Lin SJ (2009). Phthalate exposure in girls during early puberty. J Pediatr Endocrinol Metab.

[b8-ehp-118-1039] Fang H, Tong W, Perkins R, Soto AM, Prechtl NV, Sheehan DM (2000). Quantitative comparisons of *in vitro* assays for estrogenic activities. Environ Health Perspect.

[b9-ehp-118-1039] Greenland S, Pearl J, Robins JM (1999). Causal diagrams for epidemiologic research. Epidemiology.

[b10-ehp-118-1039] Hauser R, Duty S, Godfrey-Bailey L, Calafat AM (2004). Medications as a source of human exposure to phthalates. Environ Health Perspect.

[b11-ehp-118-1039] Heavner DL, Morgan WT, Sears SB, Richardson JD, Byrd GD, Ogden MW (2006). Effect of creatinine and specific gravity normalization techniques on xenobiotic biomarkers in smokers’ spot and 24-h urines. J Pharm Biomed Anal.

[b12-ehp-118-1039] Horn-Ross PL (1995). Phytoestrogens, body composition, and breast cancer. Cancer Causes Control.

[b13-ehp-118-1039] Jacobson-Dickman E, Lee MM (2009). The influence of endocrine disruptors on pubertal timing. Curr Opin Endocrinol Diabetes Obes.

[b14-ehp-118-1039] Kaplowitz PB (2008). Link between body fat and the timing of puberty. Pediatrics.

[b15-ehp-118-1039] Kato K, Silva MJ, Needham LL, Calafat AM (2005). Determination of 16 phthalate metabolites in urine using automated sample preparation and on-line preconcentration/high-performance liquid chromatography/tandem mass spectrometry. Anal Chem.

[b16-ehp-118-1039] Miller RC, Brindle E, Holman DJ, Shofer J, Klein NA, Soules MR (2004). Comparison of specific gravity and creatinine for normalizing urinary reproductive hormone concentrations. Clin Chem.

[b17-ehp-118-1039] National Science Foundation (2008). Phthalates and Cumulative Risk Assessment: The Tasks Ahead.

[b18-ehp-118-1039] Rasier G, Toppari J, Parent AS, Bourguignon JP (2006). Female sexual maturation and reproduction after prepubertal exposure to estrogens and endocrine disrupting chemicals: a review of rodent and human data. Mol Cell Endocrinol.

[b19-ehp-118-1039] Rybak ME, Parker DL, Pfeiffer CM (2008). Determination of urinary phytoestrogens by HPLC-MS/MS: a comparison of atmospheric pressure chemical ionization (APCI) and electrospray ionization (ESI). J Chromatogr B Analyt Technol Biomed Life Sci.

[b20-ehp-118-1039] Schoeters G, Den HE, Dhooge W, van LN, Leijs M (2008). Endocrine disruptors and abnormalities of pubertal development. Basic Clin Pharmacol Toxicol.

[b21-ehp-118-1039] Selevan SG, Rice DC, Hogan KA, Euling SY, Pfahles-Hutchens A, Bethel J (2003). Blood lead concentration and delayed puberty in girls. N Engl J Med.

[b22-ehp-118-1039] Shen O, Du G, Sun H, Wu W, Jiang Y, Song L (2009). Comparison of *in vitro* hormone activities of selected phthalates using reporter gene assays. Toxicol Lett.

[b23-ehp-118-1039] Teitelbaum SL, Britton JA, Calafat AM, Ye X, Silva MJ, Reidy JA (2008). Temporal variability in urinary concentrations of phthalate metabolites, phytoestrogens and phenols among minority children in the United States. Environ Res.

[b24-ehp-118-1039] van Wieringen JC, Roede MJ, Wit JM (1985). Growth diagrams for patient care [in Dutch]. Tijdschr Kindergeneeskd.

[b25-ehp-118-1039] Weinberg CR (1993). Toward a clearer definition of confounding. Am J Epidemiol.

[b26-ehp-118-1039] Wolff MS, Britton JA, Boguski L, Hochman S, Maloney N, Serra N (2008). Environmental exposures and puberty in inner-city girls. Environ Res.

[b27-ehp-118-1039] Wolff MS, Teitelbaum SL, Windham G, Pinney SM, Britton JA, Chelimo C (2007). Pilot study of urinary biomarkers of phytoestrogens, phthalates, and phenols in girls. Environ Health Perspect.

[b28-ehp-118-1039] Wu T, Buck GM, Mendola P (2003). Blood lead levels and sexual maturation in U.S. girls: the Third National Health and Nutrition Examination Survey, 1988–1994. Environ Health Perspect.

[b29-ehp-118-1039] Ye X, Kuklenyik Z, Bishop AM, Needham LL, Calafat AM (2006). Quantification of the urinary concentrations of parabens in humans by on-line solid phase extraction-high performance liquid chromatography-isotope dilution tandem mass spectrometry. J Chromatogr B Analyt Technol Biomed Life Sci.

[b30-ehp-118-1039] Ye X, Kuklenyik Z, Needham LL, Calafat AM (2005). Automated on-line column-switching HPLC-MS/MS method with peak focusing for the determination of nine environmental phenols in urine. Anal Chem.

[b31-ehp-118-1039] Zou G (2004). A modified poisson regression approach to prospective studies with binary data. Am J Epidemiol.

